# Nutritional Quality of Fresh Tuber and Flour From *Arisaema schimperianum* (*Amoch*) Food Plant as Affected by Pretreatment Drying Methods

**DOI:** 10.1155/ijfo/9545629

**Published:** 2025-05-05

**Authors:** Gezahegn Abawa Zeleke, Adamu Zegeye Hadis

**Affiliations:** ^1^Department of Food Engineering, College of Engineering and Agro-Industrial Technology, Arba Minch University, Ethiopia; ^2^School of Chemical and Bio Engineering, Addis Ababa Institute of Technology, Addis Ababa University, Ethiopia; ^3^School of Chemical and Bioengineering, Institute of Technology, Addis Ababa University, Ethiopia

**Keywords:** antinutritional, *Arisaema*, drying, nutritional composition

## Abstract

The present study analyzed the nutritional composition, antinutrient, and mineral composition of fresh *Arisaema schimperianum* tuber and flour popularly consumed in southern Ethiopia. Tubers and flour were subjected to laboratory analysis for moisture, crude protein, total ash, crude fiber, crude fat, total carbohydrate, gross energy, and minerals: Ca, Fe, Mn, Zn, and P. Antinutritional factors (phytate, oxalate, and tannin) were determined following standard procedures. The flours were prepared separately from sun-dried (SD), freeze-dried (FD), and oven-dried (OD) tubers. Fresh tubers and flour from SD, FD, and OD *Arisaema schimperianum* had the following compositions: moisture content of 86.77%, 13.19%, 13.95%, and 11.29%, respectively; crude protein of 1.44%, 2.22%, 2.9%, and 1.93%, respectively; total ash of 4.4%, 5.85%, 5.45%, and 6.18%, respectively; crude fiber of 2.36%, 2.51%, 2.72%, and 3.11%, respectively; crude fat of 1.93%, 2.37%, 2.68%, and 3.23%, respectively; total carbohydrate of 5.6%, 76.06%, 75.05%, and 78.11%, respectively; gross energy of 44.97%, 334.36%, 312.08%, and 349.23%, respectively. The Ca content (mg/100 g) of fresh and flour (SD, FD, and OD) was found to be 94.15, 44.26, 60.00, and 50.53, respectively; the Fe content (mg/100 g) was 15.84, 6.17, 9.37, and 6.05; the Zn content was 7.64, 5.20, 6.14, and 6.13; the P content was 56.11, 43.02, 48.44, and 40.12; and the Mn content was 1.91, 1.49, 1.76, and 0.97. No significant differences in Zn content were observed between the OD and FD products. The phytate contents (mg/100 g) of the fresh tuber and SD, FD, and OD flour were 32.18, 18.98, 20.26, and 15.51, respectively. Oxalate (mg/100 g) was 22.05, 6.66, 9.96, and 6.19, while tannin (mg/100 g) was found to be 14.03, 7.39, 8.11, and 6.42, respectively. In conclusion, fresh tuber and flour from *Arisaema schimperianum* are nutritious and rich in phosphorus, calcium, and iron. However, drying before milling reduced the mineral content and antinutritional factors while increasing the proximate values, except for the moisture content.

## 1. Introduction

Wild plants are consumed less for edible purposes due to the presence of some toxic and poisonous components [1]. They have various nutritional and medicinal properties [2–4] to ensure the safety of humans from different diseases. *Arisaema schimperianum* is a good example of a wild edible plant found in the Araceae family, a perennial tuber plant that grows up to a height of 20–75 cm. It is native to Afghanistan, China, India, Kashmir, Nepal, Pakistan, Sudan, Ethiopia, and Uganda. The genus *Arisaema* has about 150 species [5]. It is species-ordinarily male when it is little, in its first growing stage, and female or bisexual when it is large, matured, with a single plant capable of changing sex depending on its nutritional life [6]. *Arisaema schimperianum is* a perennial tuber plant that provides daily calories [7] and is widely consumed in the southern part of Ethiopia [8]; it is a seasonal crop that grows as a weed in almost all places of the Gamo highland [9]. Amochi yields up to 12 tons per hectare [10] (Gedebo, unpubl.), which is comparable to the yields of the main season crops of the area [11].

*Arisaema schimperianum* has many names that vary from region to region in Ethiopia; it is called *Qoltso* in southern Ethiopia (Gofa) and *Amoch* in the Amharic language [12]. *Arisaema* is likewise referred to as a “hard-time crop,” “the mother of the poor,” and “crop of the poor” [9]. The plant is considered food for hunger or famine and is reported to have more nutritional value than conventional food plants [13]. *Arisaema* plays an important role in maintaining livelihood security and closing food gaps during droughts or shortages for many people in developing countries [14] like Ethiopia. The finished product of *Arisaema* can be converted into different types of local food or, most often, mixed with *qocho*, which is one of the staple foods of the study area, barley, or other food raw materials and made into a variety of foods [9]. The objective of the present study is to analyze the nutritional composition, antinutritional factors, and mineral content of the fresh tubers and processed flour of wild edible *Arisaema schimperianum.*

## 2. Materials and Methods

### 2.1. Description of the Area

*Arisaema* tubers were collected from two locations, namely, Giyassa and Dalbansa, Gamo, Ethiopia, which are located at an altitude ranging from 1700 to 2400 m above sea level, 6° 11.5⁣′ N latitude, and 37° 29.5⁣′ E longitude. According to meteorological measurements, the minimum temperature of the area is 19°C, and the maximum temperature is 25°C. These sites were considered research sites due to the potential use of *Arisaema* as a food crop by the local population.

### 2.2. Sample Collection and Preparation

Four kilograms of *Arisaema* tuber plant material were transported to the Department of Chemistry of the Arbaminch University for preliminary processing: washing with distilled water, size reduction, using three drying methods (sun drying, freeze drying, and oven drying) and fresh tuber in triplicate, a total of four sample sizes, and pulverizing with a mechanical grinder (high-speed multifunctional crusher). After using a 0.5-mm sieve, the flour was packed in transparent polyethylene zip-lock bags and transported to the Addis Ababa University Center for Food Science and Nutrition for nutritional, antinutritional, and menial analysis.

### 2.3. Drying Methods

**Figure 1:**
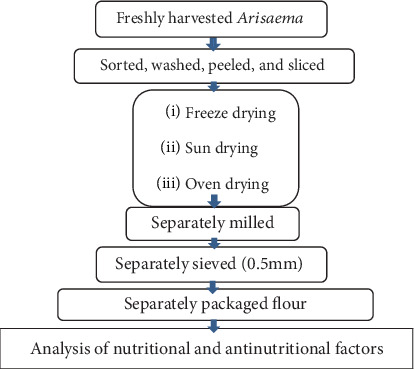
*Arisaema* flour preparation flow sheet.

The freeze-drying procedure was carried out as described in [15], with slight modifications, using a freeze dryer (Alpha 1-2 LD plus, German). One hundred grams of cleaned *Arisaema* tubers was manually peeled and thinly cut into 2-mm thick slices. The slices were placed in zip-lock bags pierced with holes and frozen in a deep freezer at −21°C for 24 h before being placed in a freeze dryer. The holes were allowed to regulate the temperature and pressure inside and outside the zip-lock bags during the drying process. Primary drying was carried out at −41°C and 0.11 mb pressure, while final drying was carried out at −47°C and 0.055 mb. The freeze-drying process lasted 48 h in total. For open-air drying, *Arisaema* tuber slices were placed on a tray and dried in the sun for 5–7 days continuously for 8 h, starting at 9:00 a.m. to 5:00 p.m. at about 33°C and relative humidity of 58%. Similarly, thin-sliced *Arisaema schimperianum* tubers were placed in an oven dryer (30°C–106°C Mommel, China) at 60°C for 48 h. Subsequently, the three types of dried samples were converted into flour and analyzed separately (Figure 1).

### 2.4. Nutritional Analysis

The fresh tubers and flour were then subjected to proximate, antinutritional, and mineral analyses, according to standard procedures [16, 17].

#### 2.4.1. Carbohydrates

The total carbohydrate content was obtained by subtracting the determined values of protein, fat, moisture, and ash from 100%.

#### 2.4.2. Determination of Gross Energy

The gross energy was determined by calculating the fat content, carbohydrates, and protein using Atwater's conversion factors: 16.7 kJ/g (4 kcal/g) for protein, 37.4 kJ/g (9 kcal/g) for fat, and 16.7 kJ/g (4 kcal/g) for carbohydrates and expressed in calories [18].

#### 2.4.3. Total Starch

Fresh tubers were washed, peeled, cut into small pieces, immersed in a 0.2% NaOH aqueous solution for 5 h, and then homogenized using a blender. The mixture was squeezed through five layers of cloth and filtered using 100, 200, and 300 mesh sieves sequentially. The starch was washed five times with 0.2% NaOH aqueous solution, three times with distilled water, and two times with anhydrous ethanol by centrifugation. Finally, the precipitated starch was dried at 35°C for 24 h, ground to powder, and passed through a 100-mesh sieve. The starch yield was determined based on 400 g of peeled and mixed *Arisaema*. The starch content in the flour was determined following the method of [19].

(1) $$\begin{array}{c} \text{Starch yield }\left(\%\right)=\left(\frac{\text{mass of dried sarch}}{\;400\text{ g}}*100\right) \end{array}$$

### 2.5. Atomic Absorption Spectroscopy

#### 2.5.1. Minerals Determination

The mineral content (Ca, Fe, Mn, Zn, and P) of the *Arisaema* tuber was determined by atomic absorption spectroscopy. The triple acid digestion method [20] was used; 2 g of sample was mixed with 24 cm^3^ of concentrated nitric acid (HNO₃), sulfuric acid (H₂SO₄), and 60% perchloric acid (HClO₄) (9:2:1 v/v), digested for 10 min to a clear solution, cooled, and transferred to a 50-cm^3^ volumetric flask and exceeded the mark with deionized water. The mineral content was determined by atomic absorption spectroscopy.

### 2.6. Antinutrient Determination

#### 2.6.1. Determination of Oxalates

Oxalate was determined as 75 mL of 3.0 M H₂SO₄, which was added to 1 g of each ground sample and stirred intermittently with a magnetic stirrer for approximately 1 h and then filtered. Twenty five milliliters of a filtrate sample (extract) was collected and titrated while hot (80°C) against a solution of 0.05 M KMnO₄ to the point where a faint pink color appeared that was persistent for at least 30 s [21, 22].

(2) $$\begin{array}{c} \text{Oxalate content }\left(\frac{\text{mg}}{100\text{g}}\right)=\left(\frac{T*\left[V\text{me}\right]\left[\text{DF}\right]*2.4*10^{2}\;}{\text{ ME}*\text{MF}}*100\right), \end{array}$$

where *T* is the KMnO_₄_, Vme is the equivalent volume mass (that is, 1 mL of 0.05 M KMnO_₄_ solution is equivalent to 0.00225 g of anhydrous oxalic acid), and DF is the dilution factor,

(3) $$\begin{array}{c} \left(\frac{\text{VT}}{A}\right), \end{array}$$

where VT is the total volume of filtrate (75 mL), *A* is the aliquot used (25 mL), ME is the equivalent molar KMnO₄, and MF is the weight of sample used.

#### 2.6.2. Determination of Phytates

Four grams of each sample was soaked in 100 mL of 2% HCl for 5 h and filtered. Twenty-five milliliters of the filtrate was measured in a 5-mL conical flask of 0.3% ammonium thiocyanate solution (NH₄SCN) as an indicator, and 53.5 mL of distilled water was also added to reach a pH of 3.5. The mixture was titrated with a ferric chloride solution (FeCl₃) until a brownish-yellow color persisted for 5 min. The phytate content (mg/100 g) was then calculated [23].

(4) $$\begin{array}{c} \text{Phytate content}=\left(\frac{T*0.195*3.55}{94.5}*100\right), \end{array}$$

where *T* is the titer, and 0.195, 3.55, and 94.5 are constants.

#### 2.6.3. Quantitative Estimation of Tannin

Two to three drops of 5% (w/v) ferric chloride aqueous solution were added to 1 mL of extract to observe the formation of a green precipitate, indicating the presence of tannins in the sample. Quantitative estimation of tannin was performed by titration of the extract with standard potassium (K) permanganate solution following the method of AOAC [24]. Briefly, 5 mL of extract aliquot was mixed with 375 mL of distilled water and 12.5 mL of indigo carmine solution; this mixture was titrated against the KMnO4 solution (“*Y*” milliliters). During the titration process, the indigo carmine's blue color changed through several colors until ending up as yellow with a slight pink undertone at the edges. That was considered the endpoint. KMnO₄ is used to titrate total tannin plus all related compounds. To determine the volume of KMnO₄ (“*X*” milliliters ) used to titrate the nontannin compounds (related), another 50 mL extract aliquot was mixed with 25 mL of gelatin solution for 1 h in saturated NaCl solution. Once the mixture cooled, we created the solution using 5 g of powdered kaolin, 50 mL of acidic NaCl solution (25 mL of concentrated H₂SO₄ added to 975 mL of saturated NaCl solution), and 1 L of saturated NaCl. The mixture was heated until the gelatin had dissolved. After 15 min of shaking the mixture, 12.5 mL of the filtrate was mixed with 375 mL of distilled water and the same volume of indigo carmine solution. The combination was then filtered through Whatman No. One filter paper. This mixture was titrated against the KMnO₄ solution until the color changed to a faint pink as earlier. The calculation of *Y* and *X* values was applied using the volume of KMnO₄ titrate of true tannin. The tannin concentration was estimated using the following relationship: 0.595 mL of 0.1 N oxalic acid is equal to 1 mL of normal KMnO₄ solution. 0.1 N oxalic acid in 1 ml equals 0.0042 g of tannin.

### 2.7. Data Analysis

A completely randomized design (CRD) was used for the experiment. Samples were investigated in triplicate, and data was subjected to analysis of variance (ANOVA). The mean was compared using Duncan's multiple range test (DMRT) using SPSS statistical software (SPSS 29.0 for Windows, SPSS Inc., Illinois, United States).

## 3. Results and Discussion

### 3.1. Nutritional Analysis

#### 3.1.1. Effects of Drying Techniques on the Nutritional Composition of *Arisaema* Flour

The nutritional composition of *Arisaema* flour exposed to various drying methods is introduced in Table 1. There was a significant difference (*p* < 0.05) in moisture content caused by drying techniques. The moisture content of the fresh samples was 86.77% and 13.45% in the freeze-dried, while the lower values were recorded in the oven-dried (11.29%) and in the sun-dried (13.19%), all of the rest within the same column differed significantly from each other. The fat substance would improve the flavor-holding capacity of the flour sample, and the samples would be suitable for some fat-soluble vitamins. Fiber is required for easy bowel transit of foods, reduced calorie consumption, and reduced incidence of diabetes. The highest crude protein value was recorded in FD (2.97%), and the lowest was recorded in OD (1.93%). Freeze-drying was found to preserve the protein content of *Arisaema* more than other drying methods, and this could be because freeze-drying applies low pressure and low temperature, leaving the cellular structure of *Arisaema* intact [25–27]. Total ash was highest in OD (6.18%) and lowest in FD (5.45%). The carbohydrate content ranged from 75.05% to 78.11%, while the highest energy value was recorded in OD (349.23 kcal/100 g) and the lowest in FD (312.08 kcal/100 g). The reduced moisture content due to dehydration has been reported to have an inverse relationship with the carbohydrate, ash, and fiber content [28]. *Arisaema* tuber starch had 26.80%, which shows they are good sources of starch.

### 3.2. Mineral Composition

*Arisaema schimperianum* has a mineral composition shown in Table 2 that is nutritionally significant for minerals when compared to the standard recommended dietary allowance (RDA) per 100 g. Arisaema contained an ample amount of nutrients when compared to the standard RDA. The phosphorus content in common edible plants varies from 32 to 138 mg/100 g [29]; *Arisaema schimperianum* fulfills this requirement and can be a potential source of phosphorus. The values of iron and zinc were comparable to the standard, whereas the values for manganese and calcium (Ca) were moderately low. As indicated in Table 2, all elements—Ca, Fe, P, Zn, and Mn—were significantly reduced with different drying treatments (*p* < 0.05), compared to fresh arisaema except Zn by FD and OD. But Zn did not show significantly different (*p* < 0.05) values in the FD and OD of the *Arisaema* tuber flour. The reduction in mineral content during the drying process can be attributed to oxidation that disintegrates the mineral compound [30]. Comparable studies by [31], following fruit drying, zinc levels decreased; fresh fruit had the highest levels, while improved solar drying had the lowest.

The highest zinc decrease was observed with enhanced solar drying [31]. Similar to zinc, all of the dried samples had lower levels of iron than the fresh fruit [31]. After drying in the open sun, pumpkin fruit's Ca levels were likewise significantly lowered [31]. As per [32], there was a noticeable pattern of increased mineral content in FD as opposed to OD; the average levels of Ca and K were high, ranging from 895.7 and 1035 mg/100 g DM for OD and FD, respectively. They also had higher levels of phosphorus (652.31–685.9 mg/100 g DM for OD and FD, respectively) and magnesium (145 and 161 mg/100 g DM for OD and FD, respectively). According to [33], the RDA of iron for adults, children, and babies ranges from 6 to 15 mg/kg. According to the current study, the iron content of *Arisaema schimperianum* ranged from 6.17 to 15.84 mg/kg, indicating that it may offer a substantial amount of iron when ingested. The studies' manganese concentration (01.49–1.91) was lower, most likely as a result of variations in the *Arisaema schimperianum* genetic composition.

They are good sources of zinc, and hence, meals based on them, according to the current study, contained zinc, which ranged from 5.20 to 7.64 mg/kg. In comparison to other drying methods, the current study's results from freeze drying showed a higher mineral concentration; this is consistent with research by [34]. More research is necessary to determine how drying techniques affect the mineral and antinutrient levels in Arisaema tuber flour. Many traditional African vegetables have varying mineral contents depending on their maturity stage [35, 36]. The greatest decline in mineral content (up to 97%) occurs during maturation, according to [37]; the largest decreases are recorded by Zn, Fe, and Mn (88%, 85%, and 81%, respectively), followed by Ca and P (70% and 70% decrease, respectively).

Over 200% of the fruit's Ca was lost during drying [31]. According to mineral results, fresh fruit typically had higher concentrations of each mineral, while drying decreased mineral levels [31]. Leaching during washing or boiling, as well as heat activities, can cause the loss of nutrients (minerals) [38].

### 3.3. Antinutrient Content

**Figure 2:**
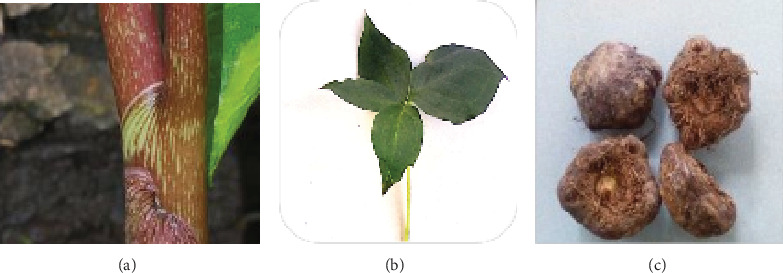
*Arisaema schimperianum* plant: (a) stem, (b) palmate leaves, and (c) tuber.

The effects of some drying methods on the antinutritional composition of *Arisaema* tuber flour revealed variations in its constituents. The results of the tannin, phytate, and oxalate content in dried tubers are presented in Table 3. The FD had the highest composition of phytate contents, whereas the oven-dried sample had the lowest. However, regardless of the drying method, phytate was the most abundant, while tannin was the least abundant phytoconstituent in Arisaema flour. The quantity of antinutrient compounds in *Arisaema* appeared to decrease dramatically as the drying temperature increased; it was observed that the different drying methods were able to decrease the amount of all oxalate, tannin, and phytate due to the high heat intensity, which disrupts the cells of the arisaema, causing it to vaporize as compared to the fresh arisaema. Similar research by [39] showed that the fresh sepals had the highest amount of oxalate; drying methods tend to decrease antinutrient levels in food [40]. The research finding on the phytate content of the fresh *Arisaema* was higher than that of the oven-, sun-, and freeze-dried, which is supported by [39], which observed that the drying methods were able to reduce the phytate level in the fresh to an appreciable level as stated by making them less adverse to human health. Images of the stem, leaves, and tubers of the *Arisaema schimperianum* plant are shown in Figure 2.

## 4. Conclusions

*Arisaema schemperianum* tubers contribute significantly to the dietary intake of a research area community (Arbaminch area) for use in times of seasonal food shortages to fill a food gap and to regularly improve the supply of staple foods and for use as emergency food during a famine. *Arisaema* tuber and its flour are nutritious and play a role in ensuring food security in the region. Predried procedures affect the concentration and bioavailability of some essential constituents of the crop. In general, there was a reduction in all nutritional compositions, antinutrients, and minerals during drying. The mineral composition of *Arisaema schimperianum* demonstrates its potential as a source of essential nutrients, particularly phosphorus, iron, and zinc, with some variations due to drying methods. Drying significantly reduced the mineral content, likely due to oxidation, though freeze drying maintained higher mineral levels compared to other methods. Antinutrient content, including phytate, oxalate, and tannin, was also affected by drying, with higher temperatures leading to a reduction in these compounds. These findings highlight the importance of drying methods in preserving both the nutritional and antinutrient content of *Arisaema schimperianum*, suggesting that freeze drying may be the most effective method for maintaining its nutritional quality. Further research is needed to explore the impact of drying techniques on nutrient and antinutrient levels.

## Figures and Tables

**Table 1:** Nutritional composition (% dry weight) of *Arisaema* subjected to fresh and various drying methods.

**Methods**	**MC content (g/100** g**)**	**Crude protein (g/100 g)**	**Crude fat (g/100 g)**	**Total ash (g/100 g)**	**Crude fiber (g/100 g)**	**Total CHO (g/100 g)**	**GE (kcal/100 g)**
R	86.77 ± 0.036^a^	1.44 ± 0.026^d^	1.93 ± 0.037^d^	4.40 ± 0.025^d^	2.36 ± 0.031^d^	5.6 ± 0.346^d^	44.97 ± 0.026^d^
OD	11.29 ± 0.300^c^	1.93 ± 0.036^c^	3.23 ± 0.176^a^	6.18 ± 0.099^a^	3.11 ± 0.026^a^	78.11 ± 0.026^a^	349.23 ± 0.046^a^
FD	13.45 ± 0.172^b^	2.97 ± 0.026^a^	2.68 ± 0.202^b^	5.45 ± 0.008^c^	2.72 ± 0.075^b^	75.05 ± 0.026^c^	312.08 ± 0.056^c^
SD	13.19 ± 0.026^b^	2.22 ± 0.026^b^	2.37 ± 0.029^c^	5.85 ± 0.150^b^	2.51 ± 0.045^c^	76.06 ± 0.026^b^	334.36 ± 0.036^b^

*Note:* The mean values in the same column that are not followed by the same letter are significantly different (*p* < 0.05, *n* = 3). The values of fresh tubers have been converted to a dry weight basis. CHO = carbohydrate, R = fresh tuber.

Abbreviations: FD = freeze-dried, GE = gross energy, MC = moisture content, OD = oven-dried, SD = sun-dried.

**Table 2:** Mineral content (mg/100 g) of *Arisaema schimperianum* (*Amoch*) tuber flour and fresh.

**Drying methods**	**Minerals** ^**∗∗**^				
	**P**	**Ca**	**Fe**	**Zn**	**Mn**
R	56.11 ± 0.026^a^	94.15 ± 0.036^a^	15.84 ± 0.046^a^	7.64 ± 0.044^a^	1.91 ± 0.01^a^
OD	40.12 ± 0.036^d^	50.53 ± 0.061^c^	6.05 ± 0.044^d^	6.13 ± 0.036^b^	0.97 ± 0.026^d^
FD	48.44 ± 0.046^b^	60.00 ± 0.044^b^	9.37 ± 0.017^b^	6.14 ± 0.026^b^	1.76 ± 0.026^b^
SD	43.02 ± 0.01^c^	44.26 ± 0.036^d^	6.17 ± 0.2^c^	5.20 ± 0.02^c^	1.49 ± 0.036^c^
RDA	300–4700	1000–3000	0.27–27	4–40	30–410

*Note:* The mean values in the same column that are not followed by the same letter are significantly different (*p* < 0.05, *n* = 3). The values of the fresh tuber have been converted to a dry weight basis. R = fresh tuber.

Abbreviations: FD = freeze-dried, OD = oven-dried, SD = sun-dried.

∗∗The listed mineral values (P, Ca, Fe, Zn, and Mn) are compared against the recommended dietary allowance (RDA) values shown in the last row. Minerals refer to the selected elements (phosphorus, calcium, iron, zinc, and manganese) whose measured concentrations in each drying method are evaluated relative to their RDA ranges.

**Table 3:** Effects of drying on antinutritional constituents of *Arisaema schimperianum.*

**Drying methods**	**Total oxalate**	**Phytate**	**Tannin**
R	22.05 ± 0.036^a^	32.18 ± 0.01^a^	14.03 ± 0.036^a^
OD	6.19 ± 0.041^d^	15.51 ± 0.015^d^	6.42 ± 0.026^d^
FD	9.96 ± 0.036^b^	20.26 ± 0.026^b^	8.11 ± 0.026^b^
SD	6.66 ± 0.017^c^	18.98 ± 0.017^c^	7.39 ± 0.026^c^

*Note:* The mean values in the same column that are not followed by the same letter are significantly different (*p* < 0.05, *n* = 3). The values of fresh tubers have been converted to the basis of dry weight. R = fresh tuber.

Abbreviations: FD = freeze-dried, OD = oven-dried, SD = sun-dried.

## Data Availability

The materials described in the manuscript, including all relevant raw data, will be freely available to any scientist who wishes to use them for noncommercial purposes.
